# Field Evaluation of a Portable Whispering Gallery Mode Accelerometer

**DOI:** 10.3390/s18124184

**Published:** 2018-11-29

**Authors:** Ying Lia Li, P. F. Barker

**Affiliations:** Department of Physics & Astronomy, University College London, London WC1E 6BT, UK; p.barker@ucl.ac.uk

**Keywords:** accelerometer, prototype, whispering gallery mode, optomechanics, optical sensing, cavity, optical sensors, resonator, field trial

## Abstract

An accelerometer utilising the optomechanical coupling between an optical whispering gallery mode (WGM) resonance and the motion of the WGM cavity itself was prototyped and field-tested on a vehicle. We describe the assembly of this portable, battery operated sensor and the field-programmable gate array automation. Pre-trial testing using an electrodynamic shaker demonstrated linear scale-factors with <0.3% standard deviation (±6 g range where g = 9.81 ms${}^{-2}$), and a strong normalised cross-correlation coefficient (NCCC) of r${}_{\mathrm{ICP}/\mathrm{WGM}}=0.997$ when compared with an integrated circuit piezoelectric (ICP) accelerometer. A noise density of 40 μg Hz${}^{-1/2}$ was obtained for frequencies of 2–7 kHz, increasing to 130 μg Hz${}^{-1/2}$ at 200 Hz, and 250 μg Hz${}^{-1/2}$ at 100 Hz. A reduction in the cross-correlation was found during the trial, r${}_{\mathrm{ICP}/\mathrm{WGM}}$ = 0.36, which we attribute to thermal fluctuations, mounting differences, and the noisy vehicle environment. The deployment of this hand-fabricated sensor, shown to operate and survive during ±60 g shocks, demonstrates important steps towards the development of a chip-scale device.

## 1. Introduction

Measurements of motion, vibration, and shock are universally required for a wide range of applications such as inertial navigation, and structural monitoring for infrastructure, health and machining [1]. Although micro-electro-mechanical systems (MEMS) dominate the consumer sensor market, there has been successful commercialisation of optical sensors such as fiber optic gyroscopes and fiber Bragg grating accelerometers [2]. In recent years, a wealth of new optical devices have emerged from the cavity optomechanics community who study the intrinsic coupling between a mechanical test-mass and an optical cavity resonance. The coupling can be dispersive and/or dissipative such that the motion causes the resonance to shift in frequency and/or change linewidth respectively [3]. These systems have similar benefits to their optical predecessors, notably an immunity to electromagnetic interference that can degrade the reliability of capacitive MEMS. Unprecedented levels of displacement sensitivity down to $10^{-18}$ m Hz${}^{-1/2}$ have been reached using cavity optomechanics [4,5], driven by techniques originating from experiments at gravitational wave observatories. Bench-top systems comprising of Fabry–Perot cavities, spherical micro-cavities that support whispering gallery mode (WGM) resonances, and chip-scale photonic crystals exploit these principles [4,5,6], and many show great promise as optical accelerometers, reaching sensitivities of ≤micro-g Hz${}^{-1/2}$ (g = 9.81 ms${}^{-2}$) [6,7,8], sufficient for detecting, for example, the acceleration of blood through the heart [9]. Subtle technical differences limit the sensitivity, known as the spectral noise density, between optomechanical and capacitive accelerometers but, in general, capacitive sensors require larger test-mass deflections and heavier proof masses to obtain a micro-g resolution, which in turn reduces the sensing bandwidth [10]. This places stringent demands on lowering parasitic and electronic noise to detect changes lower than 1 pF/g [10]. Cavity optomechanical systems, on the other hand, benefit from large single-photon optomechanical coupling strengths resulting in measurable shifts and linewidth broadening of the cavity resonance, even, in some cases, when the mechanical oscillator is displaced by its zero point motion [11]. Owing to their small size, ease of integration with industry standard components and known routes towards chip-scale fabrication [12], these sensors offer attractive commercial opportunities. In addition to the optomechanical coupling of WGMs to motion, these resonances also possess a dispersive interaction with temperature [13], and the rate of rotation [14,15].

We previously demonstrated an optomechanical accelerometer that uses optical WGMs to detect the motion of the optical cavity itself, reaching a noise density of 4.5 μg Hz${}^{-1/2}$ in the laboratory [7]. The sensor operates through evanescent coupling between a WGM cavity placed less than 1.8 μm away from an evanescent tapered waveguide. Deflections of the cavity in response to acceleration alter the coupling gap, creating measurable shifting and broadening of the WGM. Testing in the field is required to evaluate the WGM sensor’s utility and to understand the sensor operation, including the broadband performance. We note that a similar WGM accelerometer, partially fabricated using MEMS techniques, was also demonstrated by others [16]. However, the standard performance specifications were not reported, and the sensor was not tested outdoors. In this work, we outline the development and automation of a portable battery powered WGM accelerometer prototype. By testing the prototype on a vehicle, we, to the best of our knowledge, achieved the first inertial measurements with a cavity optomechanical system out of the laboratory environment. We also demonstrated, for the first time, that the tapered waveguide can survive shocks of ±60 g, which would be of interest for many WGM [5,7,13,17,18,19,20,21,22] and photonic crystal experiments [6].

## 2. Optomechanical Sensing of Acceleration Using Whispering Gallery Modes

**Figure 1:**
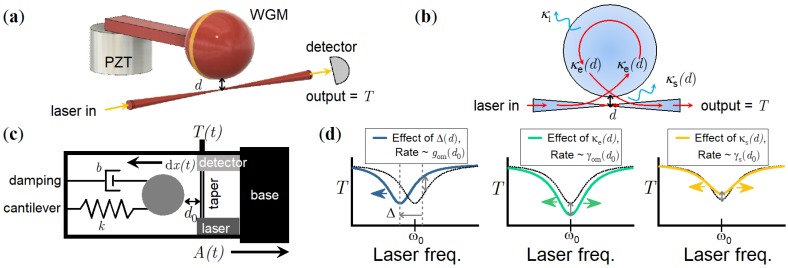
(**a**) Optical coupling between the tapered waveguide and the WGM microsphere-cantilever where *d* is the coupling gap. (**b**) Coupling light to the WGM, which alters the waveguide transmission, *T*, is determined by three optical coupling rates: the intrinsic, $\kappa_{i}$, the extrinsic, $\kappa_{e}\left(d\right)$, and a scattering rate $\kappa_{s}\left(d\right)$. There is an exponential dependence on *d* for $\kappa_{e}\left(d\right)$ and $\kappa_{s}\left(d\right)$. (**c**) Schematic of the WGM accelerometer for measuring an applied acceleration, A(t), in the *x*-axis that results in a microsphere-cantilever deflection of -dx(t) about the null-position $d_{0}$. (**d**) Three optomechanical coupling rates define the WGM transduction when $d_{0}$ changes due to the motion of the microsphere-cantilever: $g_{om}$ (dispersive), $\gamma_{om}$ (dissipative), and $\gamma_{s}$ (scattering).

The sensor consists of a microsphere cavity formed by melting the tip of a stripped rectangular core optical fiber (CeramOptec 1406R66X200R31) with a CO${}_{2}$ laser. The microsphere remained attached to the stem, which was then clamped, forming a microsphere-cantilever. A tapered optical waveguide was used for coupling light to the WGMs in the microsphere via evanescent coupling. This waveguide was fabricated by heating standard cylindrical optical fiber (Corning SMF-28) with a butane torch as it was simultaneously pulled from either end to create an evanescent field around a waist of approximately 1μm. Coupling occurred when the tapered waveguide was positioned at a coupling distance, d<1.5μm, away from the microsphere, as depicted in Figure 1a.

The coupling of photons from the waveguide to the WGM and vice versa are described by coupled-mode theory [23]. The steady state WGM intracavity electromagnetic field, *a*, valid at timescales longer than the photon lifetime, is given by:

(1) $$a=\frac{\sqrt{\kappa_{e}}a_{\mathrm{in}}}{\left(i\Delta +\frac{\kappa_{i}}{2}+\frac{\kappa_{e}}{2}+\frac{\kappa_{s}}{2}\right)},$$

where $a_{\mathrm{in}}$ is defined by the laser input power, $P_{\mathrm{in}}=a_{\mathrm{in}}^{2}\hbar \omega$, and $\Delta =\omega -\omega_{0}$ is the detuning of the laser from the WGM resonance frequency $\omega_{0}$. The amount of laser power coupled into the WGM, as depicted in Figure 1b, is therefore governed by three coupling rates: the extrinsic coupling $\kappa_{e}$ that controls light transfer from waveguide to WGM and vice versa, the intrinsic coupling $\kappa_{i}$, and a scattering component $\kappa_{s}$. The microsphere material losses and surface roughness limits $\kappa_{i}$, whereas $\kappa_{s}$ accounts for optical losses that do not couple back into the waveguide [17]. The detected signal past the coupling region, at the output of the tapered waveguide, is defined by $a_{\mathrm{out}}=-a_{\mathrm{in}}+\sqrt{\kappa_{e}}a$, and can be described by a normalised transmission, $T=|\frac{a_{\mathrm{out}}}{a_{\mathrm{in}}}{|}^{2}$:

(2) $$T={\left|1-\frac{\kappa_{e}}{\frac{\kappa_{i}}{2}+\frac{\kappa_{e}}{2}+\frac{\kappa_{s}}{2}+i\Delta }\right|}^{2},$$

where the WGM linewidth is $\kappa =\kappa_{i}+\kappa_{e}+\kappa_{s}$.

The values of Δ, $\kappa_{e}$, and $\kappa_{s}$ vary non-linearly with *d* due to the exponential decay of the evanescent field, defined by a decay constant α. An exponential red-shift of $\omega_{0}$ occurs such that, without active laser locking, the detuning changes with respect to the shifted resonance, $\Delta \left(d\right)=\Delta^{*}+\Delta_{0}e^{-\alpha d}$, where $\Delta^{*}$ is the unshifted detuning at large *d*, and $\Delta_{0}$ is the maximum shift at d=0. Similarly, $\kappa_{e}\left(d\right)=\kappa_{e,0}e^{-\alpha d}$, and $\kappa_{s}\left(d\right)=\kappa_{s,0}e^{-\alpha d}$, where $\kappa_{e,0}$ and $\kappa_{s,0}$ represent the maximum linewidth broadening effects at d=0.

Using Equation (2), it is apparent that changes to *d* will create a non-linear change in *T*, such that any motion of the microsphere-cantilever which alters *d* can be inferred. The microsphere-cantilever can therefore be used as both the mechanical test-mass that responds to acceleration as well as the optical cavity that measures the resultant cantilever deflection, as shown in Figure 1c. An acceleration, A(t), in the x-direction, causes the cantilever to deflect by a distance dx(t) away from the null-position, $d_{0}$, which is defined as the equilibrium coupling distance *d* (zero applied acceleration). The change in *T* due to $d_{0}\pm$dx(t) can be approximated by optomechanical coupling rates, defined by linearising $\kappa_{e}\left(d\right)$, $\kappa_{s}\left(d\right)$, and Δ(d) about the null-position, $d_{0}$. The rate of WGM red-shift per metre of displacement is given by the optomechanical dispersive rate $g_{\mathrm{om}}\left(d_{0}\right)=\frac{d\Delta (d_{0})}{dd_{0}}$, and the broadening of the WGM linewidth is governed by the optomechanical dissipative rate $\gamma_{\mathrm{om}}\left(d_{0}\right)=\frac{d\kappa_{e}\left(d_{0}\right)}{dd_{0}}$, and the optomechanical scattering rate $\gamma_{s}\left(d_{0}\right)=\frac{d\kappa_{s}\left(d_{0}\right)}{dd_{0}}$ (Figure 1d). Note that $g_{\mathrm{om}}$, $\gamma_{\mathrm{om}}$, and $\gamma_{s}$ are only valid for small dx(t) about $d_{0}$ which limits the linear sensing range. The relative change in *T* can now be written as the sum of each optomechanical effect on the WGM [7]:

(3) $$dT\left(t\right)=\left|g_{\mathrm{om}}\frac{\partial T}{\partial \Delta }+\gamma_{\mathrm{om}}\frac{\partial T}{\partial \kappa_{e}}+\gamma_{s}\frac{\partial T}{\partial \kappa_{s}}\right|dx\left(t\right),$$

where $\frac{\partial T}{\partial \kappa_{e}}$, $\frac{\partial T}{\partial \Delta }$, and $\frac{\partial T}{\partial \kappa_{s}}$ are derived in Appendix A.

The ratio among $g_{\mathrm{om}},\gamma_{\mathrm{om}}$, and $\gamma_{s}$ defines the scale-factor at each $d_{0}$, i.e., the relative change in *T* per metre. Full details of the measurements used to find the optomechanical coupling rates can be found in [7]. We previously demonstrated that this transduction provides sufficient modulation of the transmission, *T*, to measure the thermomechanical motion around the fundamental mechanical resonance of microsphere-cantilevers at a sensitivity of 10${}^{-12}$ m Hz${}^{-1/2}$ [18]. When operated as an accelerometer as in Figure 1c, driven motion caused by an applied acceleration is measured instead. We note that this is a different transduction method to the acceleration induced shifting of WGMs through compression of the sphere [21].

## 3. Sensor Design

### 3.1. Optical Set-Up

No free space optics were used in the prototype to reduce extraneous mechanical responses and misalignment. A fiber connectorised 1550 nm wavelength distributed feedback laser was chosen as the light source with a fiber beam-splitter used for monitoring the laser output separate to the WGM signal. Although a high signal-to-noise ratio measurement is best achieved by using the detuned transmission from narrow WGMs, active laser locking methods are challenging to employ in vibrational environments. We therefore utilised a stable thermal feedback mechanism when the light is blue-detuned from the WGM resonance which counteracts frequency and power fluctuations of the laser [22,24]. Since the laser is not actively locked, the detuning changes as a function of $d_{0}$ by $\Delta \left(d_{0}\right)=\Delta^{*}+\Delta_{0}e^{-\alpha d_{0}}$. In this case, the laser is detuned by $\Delta^{*}\approx +$300 MHz from a single WGM with intrinsic full-width half-maximum (FWHM) linewidth of $\kappa_{i}=800$ MHz when the null-position is $d_{0}>1\;\mu$m. By scanning over the WGM whilst reducing $d_{0}$, we measured $\kappa_{e}\left(d_{0}\right)=6\kappa_{i}e^{-5\times 10^{6}d_{0}}$, $\kappa_{s}\left(d_{0}\right)=30\kappa_{i}e^{-5\times 10^{6}d_{0}}$, and $\Delta \left(d_{0}\right)=300\;\mathrm{MHz}+6\kappa_{i}e^{-5\times 10^{6}d_{0}}$. The ratio between the optomechanical coupling rates, $g_{\mathrm{om}}:\gamma_{\mathrm{om}}:\gamma_{s}$, is 1:1:5. We verified that the laser remains thermally locked on the blue-detuned side for all values of $d_{0}$ because the broadening rate is larger than the rate of shift for this WGM. Excitation was repeatable; an important consideration for field work where continuous power cannot be provided. Changes in ambient temperature can result in an additional red-shift of the WGM resonance at a rate of approximately 1 GHz/K [19]. This was a negligible effect for the pre-trial tests conducted in a temperature controlled laboratory and is further discussed in Section 6 for the outdoor field-trial.

### 3.2. Mechanical Set-Up

**Figure 2:**
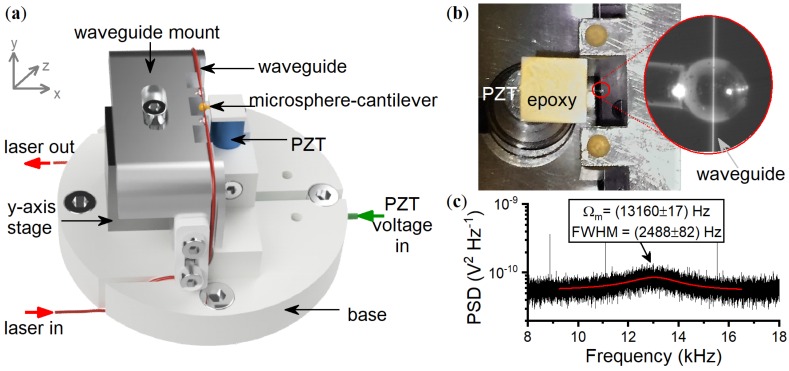
(**a**) Not-to-scale rendering of the WGM sensor components. (**b**) Photo of the microsphere-cantilever and tapered waveguide with an inset microscope image. (**c**) The power spectral density (PSD) of the WGM sensor transmission showing the fundamental mechanical mode of the microsphere-cantilever at 13.16 kHz.

The prototype was not fabricated using MEMS methods and therefore we manually aligned the waveguide and microsphere-cantilever with respect to each other. Since $d_{0}$ must be adjusted to $d_{0}<1.8\;\mu$m during operation, one cannot eliminate every translation stage or mount which could introduce unwanted mechanical responses. Therefore, at a minimum, one manual lockable translation stage is required for rough alignment and a piezostack (PZT) is then used for fine tuning $d_{0}$ in the field. The total mechanical elements are a base plate, a PZT for mounting the microsphere-cantilever, a mount for the tapered waveguide, and a 1-D manual stage for the taper mount. The base plate, specially designed to firmly secure the other pieces and minimise lateral motions, is shown in Figure 2a. The tapered waveguide is epoxied to its mount in four places: close to the taper region providing an overhang of 1 cm (Figure 2b) and then further along the mount for additional support. The remaining length of optical fiber, past the tapered region, is supported by the curved edges of the taper mount that extends to the base plate. The curved edge has a radius greater than the minimum fiber bend radius and all fibers are secured to avoid strain, vibration, or polarisation induced optical fluctuations [13]. The microsphere-cantilever is purposely positioned below the taper waveguide, ensuring the two objects are uncoupled and far away from one another ($d_{0}>10\;\mu$m) when no voltage is supplied to the PZT. This prevents damage during transportation or rough handling. The connectorised ends of the taper waveguide (input and output), as well as the input voltage cable for the PZT, are guided through trenches on the base plate such that a cylindrical chamber can be placed on top in the style of a bell-jar. Although the sensor operates at atmospheric pressure, the seal of the chamber prevents dust contamination.

One major difference between this prototype and the accelerometer we reported previously [7] is a change in the microsphere-cantilever geometry. To minimise cross-axis effects that arise when using a symmetrical cross-section cantilever such as standard cylindrical optical fiber, a rectangular cross-section fiber was employed instead such that the stiffness is higher in the lateral axis than in the vertical. Another design consideration is the compromise between sensitivity and survival as vibrational vehicles will exert shock forces that may deflect the microsphere-cantilever enough to damage the taper waveguide. A shorter cantilever reduces this deflection by increasing the spring constant, *k*, as $k\propto L^{-3}$ where *L* is the cantilever length. The dimensions of the microsphere-cantilever used in the prototype are 240 μm × 106 μm (width × height) with a cantilever length of 2.2 mm and microsphere diameter of 350 μm. When the WGM is excited at a blue detuning of 300 MHz, the fundamental (centre-of-mass) mechanical mode of the microsphere-cantilever is seen in the power spectral density (PSD) of the transmitted light, as shown in Figure 2c. The mode frequency and FWHM, found through fitting the mechanical peak in the PSD, is $\Omega_{m}=2\pi \times \left(13.160\pm 0.017\right)$ kHz and (2.488±0.082) kHz respectively, providing a mechanical quality factor of $Q_{m}=5.3$.

The spring constant is calculated as $k=469.8\;{\mathrm{Nm}}^{-1}$ from Euler–Bernoulli theory using the equation $k=\frac{3Ewh^{3}}{12L^{3}}$ where $E=70\times 10^{9}$ Pa is the Young’s modulus of silica and *L* is the length of the cantilever with cross-section w×h. Such a stiff cantilever will therefore deflect 500 nm in response to approximately 350 g without the sphere touching the taper.

## 4. Pre-Trial Characterisation

Prior to the field-test, the prototype was characterised in a controlled laboratory environment using an electrodynamic shaker (LDS 555 by Brüel & Kjær) which accurately applies a sinusoidal shake reaching peak accelerations up to ±100 g for small loads. As a crucial first check, the DFB laser was first shaken to ±10 g to determine any intensity or frequency changes that would create a false acceleration reading. Less than 0.23% intensity modulation was measured for ±10 g with a frequency shift lower than the resolution of the calibration Fabry–Perot scanning interferometer (6 MHz).

### 4.1. Scale-Factor Calibration

**Figure 3:**
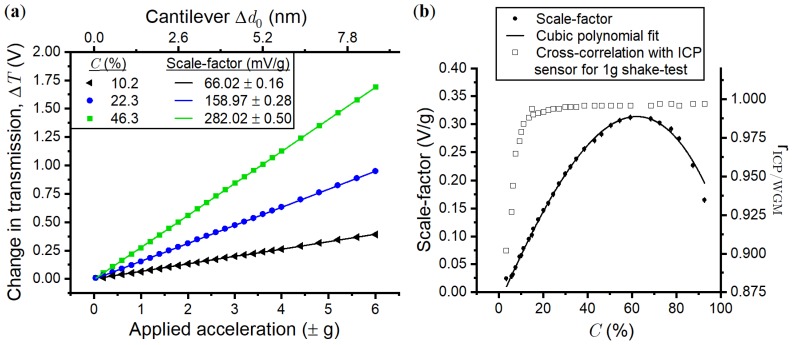
(**a**) The scale-factor is the gradient of the linear fit of the WGM response versus the applied acceleration, shown for three different $d_{0}$ defined by the coupling percentage, *C*. The corresponding cantilever deflection, $\Delta d_{0}$, is displayed as a second *x*-axis. (**b**) The scale-factor (black circles) as a function of *C* with a cubic polynomial fit (black solid line). The normalised cross-correlation coefficient, r${}_{\mathrm{ICP}/\mathrm{WGM}}$ (open squares) varies with *C* with a maximum of r${}_{\mathrm{ICP}/\mathrm{WGM}}$ = 0.997.

The scale-factor is defined as the change in transmission, ΔT, per unit of acceleration. It is found by comparing the applied acceleration from the electrodynamic shaker, $A\sin\left(\Omega_{d}t\right)$, where $\Omega_{d}$ is the shake frequency, to the WGM response ΔTsin(Ωt), such that the scale-factor equals $\frac{d(\Delta T)}{dA}$. The scale-factor unit is V/g where the voltage, V, is the output of the photodetector measuring the transmission. Previously, we showed that the scale-factor depends linearly on the input laser power, $P_{\mathrm{in}}$, and non-linearly on the null-position, $d_{0}$, due to an exponential dependence of the optomechanical coupling rates [7]. Although $d_{0}$ cannot be determined on-the-fly as it is calibrated from the point of contact when $d_{0}=0$ m, the power coupled to the WGM, $P_{c}$, can be used to infer $d_{0}$. We introduced a variable, *C*, which is the percentage of light coupled to the WGM defined by $C=\frac{P_{c}}{P_{\mathrm{in}}}\times 100\%$. Figure 3a shows ΔT versus *A* for three values of *C*, where the gradient of the linear fit determines the scale-factors. The maximum acceleration produced from the electrodynamic shaker is ±6 g at $\Omega_{d}=2\pi \times 400$ Hz, limited due to the weight of the prototype chamber (6.5 kg). The scale-factor, measured across a wide range of *C*, is plotted in Figure 3b (black circles). For an input power of $P_{\mathrm{in}}=5.5004$ V, the scale-factor varies between 0.01 V/g and 0.3 V/g as a function of *C*. Each scale factor is highly linear due to <0.3% standard deviation. A generalised analytical expression, analogous to a factory calibration, relating the scale-factor to *C*, was found by applying a best-fit polynomial to the data in Figure 3b (black solid line).

Figure 3b (open squares) also displays a measurement of the similarity between the reading from a commercial integrated circuit piezoelectric accelerometer (ICP, model 352C33 by PCB Piezotronics) and the WGM sensor output during the shake. This is defined by the linear normalised cross-correlation coefficient (NCCP), r${}_{\mathrm{ICP}/\mathrm{WGM}}$ [25], which is also used to measure the field-trial performance:

(4) $$r_{a/b}\left(m\right)=\sum_{n=0}^{N-1}\frac{a(n)}{\sqrt{\sum_{n=0}^{N-1}{\left(a\left(n\right)\right)}^{2}}}\frac{b(n-m)}{\sqrt{\sum_{i=0}^{N-1}{\left(b\left(n\right)\right)}^{2}}}=\mathrm{ifft}\left(A_{\mathrm{norm}}B_{\mathrm{norm}}^{*}\right),$$

where a(n) and b(n) are two signals of the same length *N*. The Fourier transform of the normalised signals are $A_{\mathrm{norm}}$ and $B_{\mathrm{norm}}$, respectively, with ${}^{*}$ denoting the complex conjugate. The NCCC can range −1≤0≤1, where $\left|r_{a/b}\right|<0.3$ is considered weak, $0.3<\left|r_{a/b}\right|<0.8$ is moderate, and $\left|r_{a/b}\right|>0.8$ is strong [26]. A NCCC of $r_{a/b}=1$ indicates every point in time trace a(t) is perfectly correlated with time trace b(t), i.e., a(t)=vb(t) where *v* is a constant. Note that the NCCC cannot be used to determine the underlying causes of performance degradation. Unless otherwise stated, the NCCC is provided for zero delay time, m=0 s, to analyse phase synchronicity. The NCCCs in Figure 3b (open squares) compare the ICP and WGM sensor responses to an applied acceleration of 1 g $\times \sin(\Omega_{d}t)$ as a function of *C*, noting that a low pass filter (LPF) with cut off frequency 1000 Hz was applied to both sets of data beforehand. At 3.4% coupling, r${}_{\mathrm{ICP}/\mathrm{WGM}}=0.902$, increasing to r${}_{\mathrm{ICP}/\mathrm{WGM}}=0.984$ for C= 11.2%, with r${}_{\mathrm{ICP}/\mathrm{WGM}}=0.995-0.997$ for C> 29.9%. The increase in NCCC is due to a higher signal-to-noise ratio and improvement of the thermal locking as more light is coupled with larger *C*. Poor thermal locking decreases the ability of the WGM to counteract laser frequency fluctuations which modulate the WGM signal, unrelated to acceleration. As the NCCC’s are greater than 0.9 for all *C*, and the scale-factor has good linearity, we can conclude that, for the specific case of a single frequency vibration, the sensor has negligible time delays in its response and the scale-factor variability is minimal.

### 4.2. Spectral Noise Density

**Figure 4:**
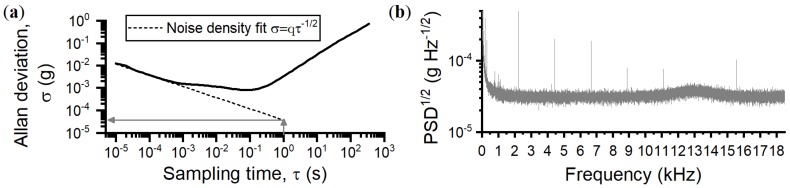
(**a**) The Allan deviation of the WGM accelerometer prototype where the dashed line is the noise density fit. The value of the fit at τ=1 s is a measure of the noise density σ=37μg Hz${}^{-1/2}$. (**b**) The noise density, as measured on the PSD, is the flat noise floor below the microsphere-cantilever mechanical peak. It increases above σ=37μg Hz${}^{-1/2}$ for frequencies below 1 kHz and frequencies around the mechanical peak. The sharp peaks are electronic noise.

The sensor’s noise density (equivalent to the velocity random walk) was found by fitting the Allan deviation with the expression $\sigma =q\tau^{-1/2}$, which defines the noise density, σ, as a function of a fitted coefficient, *q*, and sampling time, τ [27], as shown in Figure 4a. A value of σ=37μg Hz${}^{-1/2}$ was found, in good agreement with the flat noise floor of the power spectral density (PSD) in Figure 4b, valid within approximately 1–7 kHz. Outside of this frequency range, there is additional noise from the microsphere-cantilever thermomechanical motion around $\Omega_{m}$, and flicker noise at low frequency, as shown in the PSD. Between 300 Hz and 1 kHz, the noise floor is below 55 μg Hz${}^{-1/2}$, and between 50 and 300 Hz the noise floor is below 200 μg Hz${}^{-1/2}$. At frequencies below 1 Hz, σ increases sharply, as expected due to drift associated with using piezo actuation [28]; this alters $d_{0}$ and is interpreted as a false acceleration. A feedback loop is used to minimise this effect, detailed in the next section. Using finite element modeling, the first two taper modes are predicted to have resonance frequencies of 2.7 kHz and 6.8 kHz which are not transduced in the PSD due to strong damping from atmospheric pressure. Previously, we showed that the taper modes become prominent at pressures around 1 mbar [18].

The ultimate sensing limit, governed by the thermomechanical noise of the microsphere-cantilever, $a_{\mathrm{th}}$, for frequencies below the fundamental mode, is calculated using:

(5) $$a_{\mathrm{th}}=\sqrt{\frac{4k_{B}T_{0}\Omega_{m}^{3}}{kQ_{m}}},$$

where $k_{B}$ is Boltzmann’s constant and $T_{0}=300$ K is the mode temperature of the fundamental mechanical resonance. A value of $a_{\mathrm{th}}=6\;\mu$g ${\mathrm{Hz}}^{-1/2}$ was calculated using the values for the mechanical quality factor and spring constant of the fundamental mechanical mode of the microsphere-cantilever. The measured noise floor, σ, is therefore approximately six times larger than the thermomechanical noise limit. The sensor is limited by classical noise from the laser and detection chain. Techniques to reduce the detection noise for future designs are discussed in Section 7.

### 4.3. Long-Term Operation and Scale-Factor Stability

**Figure 5:**
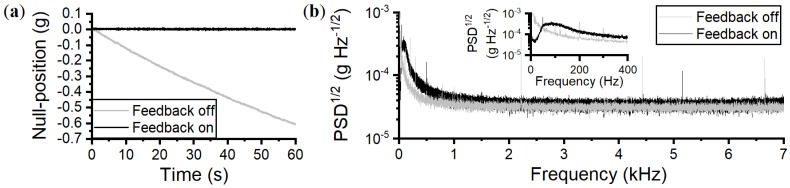
(**a**) Without feedback, creep from the PZT gradually creates a false acceleration bias as well as a change in scale-factor. To minimise scale-factor variation and enable long operation times, a simple proportional feedback was applied. (**b**) The PSD of the WGM signal with and without feedback shows the reduction in low frequency noise below 60 Hz but with a slight increase in the overall noise floor.

Previously we showed that the WGM accelerometer signal drifts over time [7], attributed to issues with PZT creep [28]. An offset in null-position will alter the scale-factor and introduce false DC readings, as shown in Figure 5a. For time periods exceeding 5 min, the PZT drift can cause the position of the microsphere-cantilever to move towards the taper such that the two objects touch. Due to strong Van der Waals and electrostatic forces [20], removal of the microsphere from the taper waveguide requires a larger amount of force which cannot be provided by the PZT alone, a difficult task to achieve in the field. It is therefore crucial to counteract this drift to enable long operation times whilst maintaining a constant scale-factor. A proportional feedback loop was implemented. First, the signal from the WGM sensor is filtered to create the user defined set-point. The difference between the measured WGM signal and this set-point creates a proportional fixed gain feedback signal sent to the PZT. The feedback operates with a bandwidth of 0–60 Hz.

This feedback maintains the null-position, as shown in Figure 5a. However, below the feedback cut-off of 60 Hz, the sensor cannot be used to measure accelerations [29]. Nonetheless, the PZT drift has been eliminated at the expense of a slight increase in noise; σ=40μg Hz${}^{-1/2}$ between 2 and 7 kHz, 130μg Hz${}^{-1/2}$ at 200 Hz, and 250μg Hz${}^{-1/2}$ at 100 Hz (Figure 5b).

## 5. Automation

**Figure 6:**
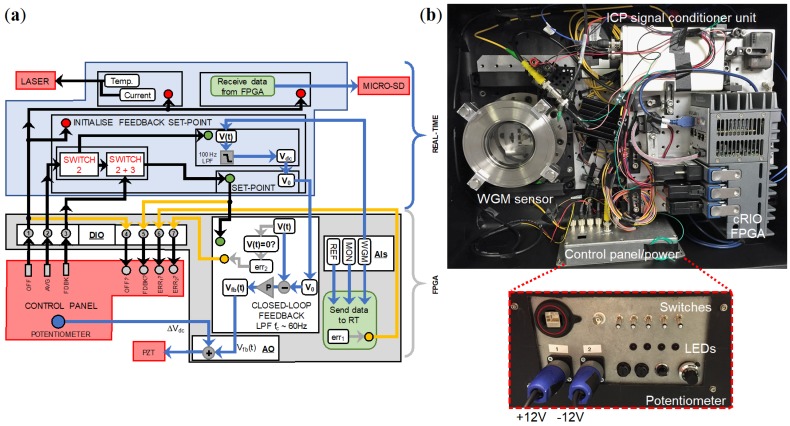
(**a**) Schematic of the automation protocol with tasks divided between the cRIO RT operating system and the embedded FPGA. (**b**) Complete prototype (portable batteries not shown) with inset photo of the outside-facing control panel.

The required level of automation is such that the user can safely operate the prototype before and after each test without any intervention during vehicle operation. The National Instruments CompactRIO model 9030 (cRIO) was chosen as the automation controller due to its resilience to vibrations up to 5 g and shocks up to 50 g. The cRIO contains an internal field-programmable gate array (FPGA, Kintex-7 70T) and a LabVIEW Real-Time operating system (RT) running on a 1.33 GHz Intel Atom Dual-Core processor, allowing for tasks to be split or communicated between the two. The automation protocol is shown in Figure 6a. The complete prototype, housed within a waterproof plastic case with dimensions 0.3×0.6×0.5 m is shown in Figure 6b, with an inset image of the outside facing control panel that distributes the battery power to the sub-components and acts as the user interface.

The prototype consumes a maximum power of 32 W which is provided by two portable rechargeable batteries supplying +12 V and −12 V. When the prototype is switched on, the cRIO initiates a start-up procedure programmed to set the laser frequency via current and temperature tuning previously calibrated in the laboratory. The laser is controlled via RS-232 communication. A portable computer oscilloscope (Picoscope 4262) is used to check the WGM whilst the laser is tuned across the mode. The laser frequency can be adjusted until the laser is ≈300 MHz detuned from resonance, with an error of approximately ±10 MHz since no active locking is used. Datalogging to a micro-SD card also begins upon power on. An analogue-to-digital converter (AI, NI9220) receives voltage signals from the photodetector recording the WGM accelerometer, another photodetector monitoring the laser output, and the signal from the ICP accelerometer previously used in the pre-trial calibration. Switches are used to initiate pre-programmed sequences and LEDs communicate whether protocols are operating smoothly. Both are controlled using a digital input/output card (DIO, NI9401) The four following errors are indicated: feedback loop is engaged or disengaged, error with the micro-SD card, microsphere-cantilever and taper waveguide touching, and prototype power off.

To initiate the feedback, the null-position is first adjusted using a potentiometer on the control panel that decreases $d_{0}$ by applying an increasing voltage. The coupling percentage, *C*, is calculated by monitoring the decreasing transmission, *T*, on the photodetector such that $C=\frac{T_{\mathrm{in}}-T_{\mathrm{set}}}{T_{\mathrm{in}}}\times 100\%$. Once the desired *C* is set, a switch is activated to isolate the slowly drifting component of *T* and another switch is engaged that creates the desired set-point and begins outputting the proportional feedback signal from a digital-to-analog module (AO, NI9263) to the PZT driver. The closed-loop gain is preset in the laboratory as described in Section 4.3. Upon switch off, an automated shut-down procedure ceases datalogging and turns off the laser. The potentiometer is manually reset and the battery power can then be disconnected.

## 6. Outdoor Field-Testing

In late 2017, the prototype was tested on a military vehicle similar to the Jackal [30], driven at speeds up to 15 m/s (approximately 33 mph) on a route containing tarmac, variable terrain grassland, and concrete curb-to-grass and vice versa transitions. Due to the varied landscape, a rich spread of vibrational frequencies was expected, creating test conditions significantly different to the single frequency shake provided by the electrodynamic shaker and is more likely to excite spurious mechanical responses. Similar issues were encountered for a cold-atom accelerometer tested on an aircraft that resulted in a 10,000 times degradation in sensitivity compared to what is achievable in the laboratory [31]. For this reason, the main goal was to validate the survival and operation of the WGM accelerometer and its sub-components. Thermal shifting of the WGM resonance was minimised through maintaining thermal equilibrium within the prototype, resulting in fluctuations of approximately ±0.2 °C. These temperature fluctuations correspond to a variation in the scale-factor, which is discussed further in Section 6.2. We dissipate heat generated by electronics by mounting all the components onto an aluminium breadboard with thermal contact to another breadboard on the outside of the case. The prototype has a warm-up time of 10 min for the laser to stabilise, assuming the prototype components are at ambient temperature. Switch off time between trial runs is kept below 3 min.

The ICP sensor previously used in pre-trial calibrations was also mounted onto the vehicle to aid analysis due to its lower noise floor of $\sigma_{\mathrm{ICP}}=3.4\;\mu$g Hz${}^{-1/2}$. The performance was analysed by three criteria: the NCCC, the ability to detect peak accelerations for impact detection applications [32,33], and the scale-factor variability.

### 6.1. Trial Data

**Figure 7:**
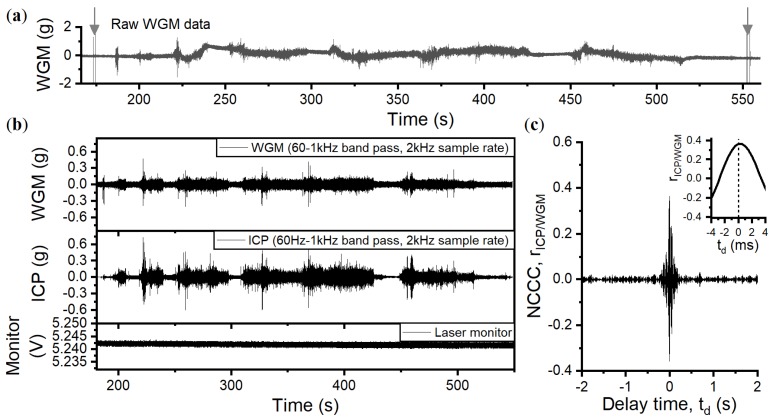
(**a**) Raw data of the WGM accelerometer during field-testing. Arrows show the hammer shocks that indicate the start and finish of the trial. (**b**) The WGM and ICP data are band pass filtered (60Hz–1 kHz) and decimated to a 2 kHz sampling rate (data below 60 Hz are unreliable due to the effect of the feedback). The laser monitor signal shows negligible changes in laser power due to acceleration. (**c**) The cross-correlation for the filtered data is r${}_{\mathrm{ICP}/\mathrm{WGM}}$ = 0.36.

The start and finish of the trial was indicated by shocks applied to the vehicle with a hammer, as marked by the arrows on the raw data displayed in Figure 7a at t = 173 s and t = 553 s. In the data presented here, the WGM sensor was positioned to measure accelerations in the direction of the driver (*x*-axis) at 10.62% coupling.

The raw data show responses at low frequency which alter the 0 g bias, and are likely related to overshooting and undershooting from the proportional feedback as it attempts to counteract slow accelerations and the PZT drift. Because of this, only data at frequencies above the feedback bandwidth of 60 Hz are evaluated. A band pass filter of 60 Hz–1 kHz was applied to the WGM and ICP data. Both datasets were then decimated to a sample rate of 2 kHz and displayed in the top and middle panels of Figure 7b. The bottom panel shows the photodetector signal monitoring the laser power output, which has negligible fluctuations in response to the applied accelerations.

Upon visual inspection, there is good sychronisation between the WGM time trace and the ICP readout, especially for shocks around bumpy terrain. The linear normalised cross-correlation coefficient (NCCC) was found to have a moderate value of r${}_{\mathrm{ICP}/\mathrm{WGM}}$ = 0.36, considerably less than the maximum NCCC of r${}_{\mathrm{ICP}/\mathrm{WGM}}=0.997$ during the electrodynamic shaker tests. During periods of low accelerations <±0.01 g, poor correlations occur due to the difference between the sensor noise floors and the signal-to-noise ratio; the ICP measures accelerations over ten times below the white noise of the WGM. The other main difference is the change in test environment. The trial has ambient temperature changes, multiple vibrations that arise from the engine, and traverses over a mixture of grassland and tarmac. Broadband vibrations could excite mechanical modes from the sensor mounts, which would not be as readily excited when applying a single frequency shake. This is especially apparent when the vehicle is driving on tarmac, where the periodic surface roughness creates an amplified sinusoidal response at time periods t = 182–218 s, t = 241–248 s, t = 300–309 s, t = 428–448 s, and t = 518–548 s. When considering non-periodic bumpy terrain like wild grassland, the NCCC is slightly improved, e.g., r${}_{\mathrm{ICP}/\mathrm{WGM}}=0.41$ between t = 310.1 s and t = 424.6 s. We discuss the effect of ambient changes in temperature in Section 6.2.

### 6.2. Dynamic Peak Response and Scale-Factor Variability

**Figure 8:**
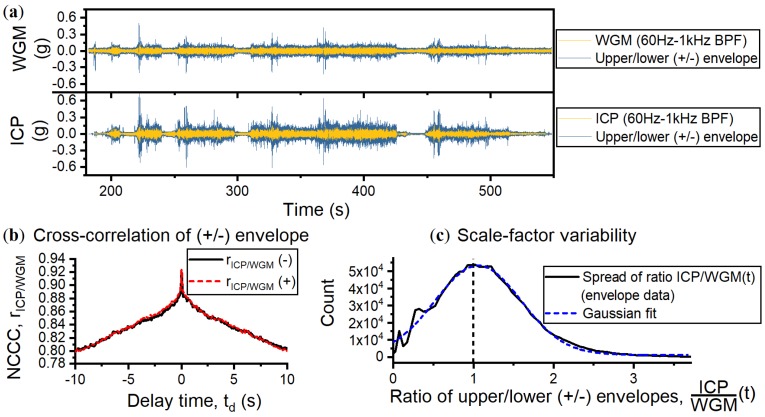
(**a**) Filtered trial data with the peak acceleration defined by upper (+) and lower (−) envelopes in blue. (**b**) Cross-correlogram of enveloped data where both envelopes have r${}_{\mathrm{ICP}/\mathrm{WGM}}=0.92$. (**c**) The distribution of the ratio $\frac{\mathrm{ICP}}{\mathrm{WGM}}\left(t\right)$ of the enveloped data only (black solid line), fitted with a Gaussian distribution (dashed blue line); the mean is $\frac{\mathrm{ICP}}{\mathrm{WGM}}\left(t\right)=1.06\pm 0.54$.

We evaluated the performance of the sensor for detecting peak accelerations, defined by the upper (+) and lower (−) envelopes of the filtered data traces in Figure 7. The two panels of Figure 8a show the data traces where the upper and lower envelopes are highlighted. The phase response of the WGM envelope data, shown in Figure 8b, is strongly correlated with the ICP with an NCCC of r${}_{\mathrm{ICP}/\mathrm{WGM}}=0.92$ for both (+) and (−) envelopes. This implies the sensor can accurately track peak accelerations such as high amplitude vibrations and impacts.

A direct comparison of the enveloped data was obtained ratiometrically by calculating $\frac{\mathrm{ICP}}{\mathrm{WGM}}\left(t\right)$ where $\frac{\mathrm{ICP}}{\mathrm{WGM}}\left(t\right)=1$ indicates both sensors output the same exact data point. We then plotted the number of data points per value of $\frac{\mathrm{ICP}}{\mathrm{WGM}}\left(t\right)$ and counted the distribution, as shown in Figure 8c. A Gaussian fit was applied showing a mean value of WGM data points are equal to 1.06 × ICP(t) with a standard deviation of 0.54. The WGM scale-factor changes by ±51% relative to the ICP, assuming the ICP scale-factor remains constant. For times when the vehicle is not traversing over tarmac, for example t = 310.1–424.6 s, the WGM signal is on average smaller than the ICP, with a mean of $\frac{\mathrm{ICP}}{\mathrm{WGM}}\left(t\right)=1.32$ and standard deviation ±0.42.

**Figure 9:**
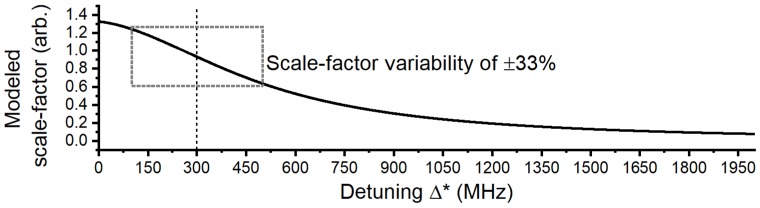
Using Equation (3), the variation of the scale-factor is modeled for ambient temperature changes of ±0.2 °C, corresponding to a thermal WGM shift of ≈±200 MHz.

Apart from spurious mechanical responses contributing to the variation in scale-factor, there are thermal fluctuations that shift the WGM resonance frequency away from the laser. This means the detuning will vary in time as $\Delta \left(t\right)=\Delta^{*}+\Delta_{0}e^{-\alpha d_{0}}+B\times {\mathrm{dT}}_{C}$, where B ≈1 GHz/°C is the rate of red-shift per degree increase in ambient temperature [13,19]. We model the effect of detuning on the scale-factor in Figure 9, using Equation (3) and the prototype optical and optomechanical coupling rates. Temperature fluctuations of ${\mathrm{dT}}_{C}=\pm 0.2$ °C, measured during the trial, are predicted to shift the WGM by approximately ±200 MHz, resulting in a scale-factor variability of ±33%, which can explain a large portion of the variability measured in Figure 8c.

## 7. Discussion

Field-testing was used to evaluate the prospects of using a WGM microsphere-cantilever as an accelerometer. We demonstrate a highly linear scale-factor of the WGM accelerometer (<0.3% fit error) and a strong cross-correlation of r${}_{\mathrm{ICP}/\mathrm{WGM}}$ = 0.997 with the ICP sensor during controlled shaker tests. This correlation was reduced during the trial, in agreement with other experiments which compare multiple accelerometers on vehicles [34]. We show for the first time that the tapered waveguide, commonly used for WGM experiments [5,7,13,17,18,19,20,21,22] and photonic crystal coupling [6], is robust and can easily survive shocks of ±60 g. A piezostack was used to tune the separation between the tapered waveguide and the WGM cavity. However, this was found to be unstable, and a different mechanism will be required for future development. This requirement could even be eliminated using MEMS fabrication.

Future developments will work towards creating a microsphere-cantilever system that is less susceptible to temperature change. Operating at low vacuum pressure will also lead to better thermal isolation, as demonstrated for a sapphire WGM accelerometer [35].

To reach the thermomechanical limited acceleration sensitivity, $a_{\mathrm{th}}=6\;\mu$g Hz${}^{-1/2}$, one must reduce all sources of detection and laser noise which currently dominate the noise floor. A balanced detection scheme and the use of a rapid laser scan across the WGM resonance instead of a fixed frequency lock could be used to reduce the signal variations with temperature. Improving $a_{\mathrm{th}}$ can be achieved by increasing the mechanical *Q*, which is difficult to obtain with the hand-fabricated microsphere-cantilevers. MEMS fabrication techniques can fabricate cantilevers with a *Q* in excess of 10,000, as well as allowing tailoring of clamping losses and test-mass material [36]. If one assumes a MEMS microsphere-cantilever with mechanical *Q* of 44,500 (at low vacuum), $\Omega_{m}=2\pi$ × 890 Hz and effective mass of $2\times 10^{-7}$ kg, an acceleration sensitivity of 10 ng Hz${}^{-1/2}$ could be achieved. This requires a displacement sensitivity of 10${}^{-15}$ m Hz${}^{-1/2}$ which has been obtained in WGM optomechanical systems [5].

## 8. Conclusions

We evaluated a microsphere-cantilever system as an optomechanical accelerometer. This sensor operates over ±6 g, with a noise density of 40 μg Hz${}^{-1/2}$ above 2 kHz, increasing to 250 μg Hz${}^{-1/2}$ at 100 Hz. We show for the first time that such an optomechanical system can be operated on a vehicle surviving ±60 g shocks. This work demonstrates the feasibility of using WGMs for sensing acceleration and indicates the future developments that would enhance its operation.

## Figures and Tables

**Figure A1:**
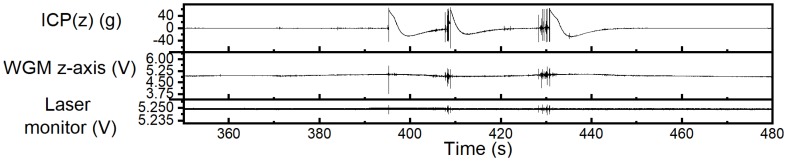
Example trial run with the ICP and WGM accelerometers measuring in the *z*-axis whilst undergoing large shocks.

## Abbreviations

The following abbreviations are used in this manuscript:

WGM	Whispering gallery mode
ICP	Integrated circuit piezoelectric
MEMS	Microelectromechanical systems
FWHM	Full width half maximum
NCCC	Normalised cross-correlation coefficient (linear)
LPF	Low pass filter
PSD	Power spectral density
FPGA	Field-programmable gate array

## Appendix A

The derivatives in Equation (3) are as follows:

(A1) $$\frac{\partial T}{\partial \kappa_{e}}=-\frac{4\left(\kappa_{i}+\kappa_{s}\right)\left(4\Delta^{2}-\kappa_{e}^{2}+{\left(\kappa_{i}+\kappa_{s}\right)}^{2}\right)}{{\left(4\Delta^{2}+\kappa^{2}\right)}^{2}},$$

(A2) $$\frac{\partial T}{\partial \Delta }=\frac{32\Delta \kappa_{e}\left(\kappa_{i}+\kappa_{s}\right)}{{\left(4\Delta^{2}+\kappa^{2}\right)}^{2}},$$

and

(A3) $$\frac{\partial T}{\partial \kappa_{s}}=-\frac{4\kappa_{e}\left(4\Delta^{2}+\kappa_{e}^{2}-{\left(\kappa_{i}+\kappa_{s}\right)}^{2}\right)}{{\left(4\Delta^{2}+\kappa^{2}\right)}^{2}},$$

where $\kappa =\kappa_{i}+\kappa_{e}+\kappa_{s}$.

## Appendix B

In Figure A1, the WGM and ICP sensors are mounted to measure accelerations in the *z*-axis (i.e., perpendicular from the ground) during a harsh trial run with additional railway tracks and obstacles that create shocks close to ±60 g. This magnitude of shock, equivalent to that of a car crash, exceeds the sensing range of the ICP sensor and introduces an additional recovery/dead time. Although the WGM sensor output becomes increasingly non-linear beyond ±6 g, the survival of the sensor is certainly impressive. During these large accelerations, an unidentified mechanical component of the laser responds to the acceleration which produces a much smaller change in light transmission than the WGM sensor (see Figure A1, bottom). Similarly, the cRIO was found to encounter errors during sustained shocks. Therefore, extra care should be taken when choosing off-the-shelf subcomponents when designing future prototypes, noting that laser operation under acceleration needs to be considered. This provides further evidence for MEMS fabrication that allows for integrated circuits and chip-scale lasers to be directly incorporated with a chip-scale sensor.

## References

[1] Barbour N., Schmidt G. (2001). Inertial sensor technology trends. IEEE Sens. J..

[2] Lee B. (2003). Review of the present status of optical fiber sensors. Opt. Fiber Technol..

[3] Aspelmeyer M., Kippenberg T.J., Marquardt F. (2014). Cavity optomechanics. Rev. Mod. Phys..

[4] Arcizet O., Cohadon P.-F., Briant T., Pinard M., Heidmann A., Mackowski J.-M., Michel C., Pinard L., Français O., Rousseau L. (2006). High-sensitivity optical monitoring of a micromechanical resonator with a quantum-limited optomechanical sensor. Phys. Rev. Lett..

[5] Schliesser A., Arcizet O., Rivière R., Anetsberger G., Kippenberg T.J. (2009). Resolved-sideband cooling and position measurement of a micromechanical oscillator close to the Heisenberg uncertainty limit. Nat. Phys..

[6] Krause A.G., Winger M., Blasius T.D., Lin Q., Painter O. (2012). A high-resolution microchip optomechanical accelerometer. Nat. Photonics.

[7] Li Y.L., Barker P.F. (2018). Characterization and testing of a micro-g whispering gallery mode optomechanical accelerometer. J. Lightw. Technol..

[8] Gerberding O., Guzmán Cervantes F., Melcher J., Pratt J.R., Taylor J.M. (2015). Optomechanical reference accelerometer. Metrologia.

[9] Meriheinä U. (2017). BCG Measurements in Beds. https://www.murata.com/en-us/products/sensor/accel/sca10h{_}11h.

[10] Krishnan G., Kshirsagar C.U., Ananthasuresh G.K., Bhat N. (2007). Micromachined high-resolution accelerometers. J. Indian Inst. Sci..

[11] Liu Y., Wang C., Zhang J., Liu Y. (2018). Cavity optomechanics: Manipulating photons and phonons towards the single-photon strong coupling. Chin. Phys. B.

[12] Miao H., Srinivasan K., Aksyuk V. (2012). A microelectromechanically controlled cavity optomechanical sensing system. New J. Phys..

[13] Xu X., Chen W., Zhao G., Li Y., Lu C., Yang L. (2018). Wireless whispering-gallery-mode sensor for thermal sensing and aerial mapping. Light Sci. Appl..

[14] Dell’Olio F., Tatoli T., Ciminelli C., Armenise M.N. (2014). Recent Advances in Miniaturized Optical Gyroscopes. J. Eur. Opt. Soc..

[15] Liang W., Ilchenko V.S., Savchenkov A.A., Dale E., Eliyahu D., Matsko A.B., Maleki L. (2017). Resonant microphotonic gyroscope. Optica.

[16] Laine J.-P., Tapalian C., Little B., Haus H. (2001). Acceleration sensor based on high-Q optical microsphere resonator and pedestal antiresonant reflecting waveguide coupler. Sens. Actuators A.

[17] Madugani R., Yang Y., Ward J.M., Le V.H., Nic Chormaic S. (2015). Optomechanical transduction and characterization of a silica microsphere pendulum via evanescent light. Appl. Phys. Lett..

[18] Li Y.L. (2016). Cooling and Sensing Using Whispering Gallery Mode Resonators. Ph.D. Thesis.

[19] Ma Q., Rossmann T., Guo Z. (2010). Whispering-gallery mode silica microsensors for cryogenic to room temperature measurement. Measur. Sci. Technol..

[20] Arnold S., Keng D., Shopova S.I., Holler S., Zurawsky W., Vollmer F. (2009). Whispering gallery mode carousel—A photonic mechanism for enhanced nanoparticle detection in biosensing. Opt. Express.

[21] Ioppolo T., Ötügen V., Fourguette D., Larocque L. (2011). Effect of acceleration on the morphology-dependent optical resonances of spherical resonators. J. Opt. Soc. Am. B.

[22] Wu Y., Ward J.M., Nic Chormaic S. (2012). Observation of thermal feedback on the optical coupling noise of a microsphere attached to a low-spring-constant cantilever. Phys. Rev. A.

[23] Haus H.A. (1984). Waves and Fields in Optoelectronics.

[24] Carmon T., Yang L., Vahala K.J. (2004). Dynamical thermal behaviour and thermal self-stability of microcavities. Opt. Express.

[25] Bendat J.S., Piersol A.G. (2011). Random Data: Analysis and Measurement Procedures.

[26] Ross S.M. (2004). Introduction to Probability and Statistics for Engineers and Scientists.

[27] Ogier E. AVAR, Hosted on the MATLAB Central File Exchange. https://uk.mathworks.com/matlabcentral/fileexchange/55765-avar.

[28] Jung H., Gweon D.-G. (2000). Creep characteristics of piezoelectric actuators. Rev. Sci. Instrum..

[29] Visioli A. (2006). Practical PID Control.

[30] Supacat Product HMT 400. https://supacat.com/products/hmt/hmt400/.

[31] Geiger R., Ménoret V., Stern G., Zahzam N., Cheinet P., Battelier B., Villing A., Moron F., Lours M., Bidel Y. (2011). Detecting inertial effects with airborne matter-wave interferometry. Nat. Commun..

[32] Duma S.M., Manoogian S.J., Bussone W.R., Brolinson P.G., Goforth M.W., Donnenwerth J.J., Greenwald R.M., Chu J.J., Crisco J.J. (2005). Analysis of real-time head accelerations in collegiate football players. Clin. J. Sport Med..

[33] Vursavus K., Ozgoven F. (2004). Determining the effects of vibration parameters and packaging method on mechanical damage in golden delicious apples. Turk. J. Agric. For..

[34] Ehsani J.P., O’Brien F., Simons-Morton B. Comparing g-force measurement between a smartphone app and an in-vehicle accelerometer. Proceedings of the Ninth International Driving Symposium on Human Factors in Driver Assessment, Training and Vehicle Design.

[35] Martin L.P., Suter J.J., Rosen M. (1994). Sapphire resonator transducer accelerometer for space gravity gradiometry. J. Phys. D Appl. Phys..

[36] Zwahlen P., Dong Y., Nguyen A.-M., Rudolf F., Stauffer J.-M., Ullah P., Ragot V. Breakthrough in high performance inertial navigation grade sigma-delta MEMS accelerometer. Proceedings of the IEEE/ION Position, Location and Navigation Symposium 2012.

